# SUMO-Targeted Ubiquitin Ligases (STUbLs) Reduce the Toxicity and Abnormal Transcriptional Activity Associated With a Mutant, Aggregation-Prone Fragment of Huntingtin

**DOI:** 10.3389/fgene.2018.00379

**Published:** 2018-09-18

**Authors:** Kentaro Ohkuni, Nagesh Pasupala, Jennifer Peek, Grace Lauren Holloway, Gloria D. Sclar, Reuben Levy-Myers, Richard E. Baker, Munira A. Basrai, Oliver Kerscher

**Affiliations:** ^1^Genetics Branch, Center for Cancer Research, National Cancer Institute, National Institutes of Health, Bethesda, MD, United States; ^2^Biology Department, College of William & Mary, Williamsburg, VA, United States; ^3^Department of Microbiology and Physiological Systems, University of Massachusetts Medical School, Worcester, MA, United States

**Keywords:** Slx5, STUbL, SUMO, ubiquitin, Htt

## Abstract

Cell viability and gene expression profiles are altered in cellular models of neurodegenerative disorders such as Huntington’s Disease (HD). Using the yeast model system, we show that the SUMO-targeted ubiquitin ligase (STUbL) Slx5 reduces the toxicity and abnormal transcriptional activity associated with a mutant, aggregation-prone fragment of huntingtin (Htt), the causative agent of HD. We demonstrate that expression of an aggregation-prone Htt construct with 103 glutamine residues (103Q), but not the non-expanded form (25Q), results in severe growth defects in *slx5*Δ and *slx8*Δ cells. Since Slx5 is a nuclear protein and because Htt expression affects gene transcription, we assessed the effect of STUbLs on the transcriptional properties of aggregation-prone Htt. Expression of Htt 25Q and 55Q fused to the Gal4 activation domain (AD) resulted in reporter gene auto-activation. Remarkably, the auto-activation of Htt constructs was abolished by expression of Slx5 fused to the Gal4 DNA-binding domain (BD-Slx5). In support of these observations, RNF4, the human ortholog of Slx5, curbs the aberrant transcriptional activity of aggregation-prone Htt in yeast and a variety of cultured human cell lines. Functionally, we find that an extra copy of *SLX5* specifically reduces Htt aggregates in the cytosol as well as chromatin-associated Htt aggregates in the nucleus. Finally, using RNA sequencing, we identified and confirmed specific targets of Htt’s transcriptional activity that are modulated by Slx5. In summary, this study of STUbLs uncovers a conserved pathway that counteracts the accumulation of aggregating, transcriptionally active Htt (and possibly other poly-glutamine expanded proteins) on chromatin in both yeast and in mammalian cells.

## Introduction

Ubiquitin and SUMO are members of a conserved family of small ubiquitin-like modifier proteins (UBLs) that can be conjugated to lysine residues of target proteins to modulate their activity, function, localization, and half-life. The conjugation of both SUMO and ubiquitin to numerous target proteins is a multi-step process and involves a cascade of similar, yet distinct E1 activating enzymes, E2 conjugating enzymes, and E3 ligases. Additionally, dedicated SUMO or ubiquitin-specific proteases render these protein modifiers conjugation competent and also aid in their deconjugation from modified proteins. As such, the dynamic conjugation and deconjugation of UBLs has key roles in cell growth and the maintenance of genome integrity and has been implicated in disease-related processes including cancer, inflammation, and neurodegeneration (Hoeller et al., 2006; Kerscher et al., 2006; Dorval and Fraser, 2007; Dasso, 2008; Liu and Shuai, 2008; Sarge and Park-Sarge, 2009; van Wijk et al., 2011; Cubeñas-Potts and Matunis, 2013). Mammalian cells express one form of ubiquitin and three forms of conjugatable SUMO (SUMO-1, SUMO-2, and SUMO-3), while budding yeast only expresses one form each of ubiquitin and SUMO (Smt3). Chains of ubiquitin can be formed through conjugation of internal lysines. Analogously, Smt3, SUMO-2, and SUMO-3 can form SUMO chains on the proteins they modify, a property not shared by SUMO-1, which lacks the internal lysines required for polymerization (Ulrich, 2008; Vertegaal, 2010). The majority of proteins that are modified with ubiquitin chains are targeted to the proteasome. In contrast, SUMO chains and hybrid SUMO-ubiquitin chains do not play a direct role in proteolytic targeting but play an important but poorly understood role in SUMO-dependent signaling and the regulation of chromatin (Guzzo et al., 2012).

STUbLs, including the heterodimeric Slx5/Slx8 in budding yeast and the RNF4 homodimer in mammalian cells, are ubiquitin E3 ligases that can specifically target and bind sumoylated proteins and facilitate their ubiquitylation. Members of this unusual subfamily of ubiquitin ligases are well conserved, contain a RING domain required for their ubiquitylation activity, and use multiple SIMs (SUMO-interacting motifs) to target sumoylated substrates (Wang et al., 2006; Kerscher, 2007; Sun et al., 2007; Uzunova et al., 2007; Xie et al., 2007; Mullen and Brill, 2008; Tatham et al., 2008; Prudden et al., 2011; Alonso et al., 2012). Not surprisingly, STUbLs play an important role in the cross-regulation of proteins that can be modified with both SUMO and ubiquitin (Perry et al., 2008; Geoffroy and Hay, 2009). Deletion of *SLX5* and *SLX8* results in the accumulation of high-molecular weight SUMO adducts and renders cells hypersensitive to DNA damage and perturbed DNA replication (Zhang et al., 2006; Prudden et al., 2007). Similarly, depletion of RNF4 sensitizes cells to DNA damage (Tatham et al., 2008; Geoffroy and Hay, 2009; Yin et al., 2012). However, several lines of evidence suggest that STUbLs also play a critical role in protein quality control. For example, Slx5/Slx8 plays a role in degrading a mutant yeast transcriptional regulator, *mot1-103*, the nuclear degradation of the SUMO E3 ligase Siz1 in mitosis, and proteolysis of centromeric histone H3 variant Cse4, and also a transcription factor, Mat α2, that is not modified with SUMO (Wang and Prelich, 2009; Westerbeck et al., 2014; Hickey and Hochstrasser, 2015; Ohkuni et al., 2016). Similarly, RNF4 has been shown to regulate the SUMO- and ubiquitin-mediated proteasomal degradation of a mutant cystic fibrosis transmembrane conductance regulator (F508del CFTR), mutant attaxin (Atxn1 82Q), and possibly the reduction of SDS-resistant aggregates of mutant huntingtin (Htt, 97QP) in the cytosol of mammalian cells (Ahner et al., 2013; Guo et al., 2014).

Mutant Htt is the causative agent of Huntington’s disease (HD), a hereditary neurodegenerative illness that affects 2.71 per 100,000 people worldwide (Pringsheim et al., 2012). The IT15 gene, first discovered in 1993, encodes the huntingtin protein (Htt) which is essential for normal development of mammals and interacts with a variety of proteins implicated in transcription, intracellular transport, and cell signaling. However, the complete extent of Htt function remains unknown (Cattaneo et al., 2005). The amino-terminus of Htt normally contains a stretch of 17–28 glutamine (Q) residues, that is expanded to more than 36Q (and sometimes over 100) in patients with HD. These amino-terminal poly-glutamine expansions form aggregates of mutant Htt that visibly accumulate in neurons and in cell culture models, including budding yeast.

It has been suggested that Htt aggregates may be neuroprotective in that they incorporate cytotoxic Htt monomers into inert cellular inclusions (Arrasate et al., 2004). However, there is also ample evidence that cellular aggregates of Htt sequester a variety of other proteins required for vesicle trafficking, cell cycle regulation, transcriptional regulation, cytoskeletal functions, cell signaling, and protein turnover, thus contributing to the demise of cells expressing these aggregation-prone proteins (Suhr et al., 2001; Bauer et al., 2012).

The majority of Htt aggregates occur in the cytosol, but there is considerable evidence that the accumulation of Htt in the nucleus enhances its toxicity [reviewed in Davies et al. (1997), Lunkes and Mandel (1997), and Benn et al. (2008)]. For example, it has recently been found that a poly-glutamine expanded Htt protein fused with a nuclear localization signal (Htt-103Q-NLS), unlike Htt-103Q, is highly toxic to Wild-type (WT) budding yeast (Wolfe et al., 2014). Interestingly, the authors of this study found that toxicity of Htt-103Q-NLS can be suppressed by overexpression of other poly-Q rich proteins including Nab3, an RNA binding protein (Wolfe et al., 2014). The poly-Q tract on Htt is known to sequester other naturally occurring proteins with poly-Q tracts. For example, there are 14 proteins with poly-Q tracts of at least 20 glutamines encoded in the yeast genome, and at least 66 genes in the human genome that have been classified as encoding poly-Q proteins (Butland et al., 2007). Proteins with poly-Q tracts are involved in a variety of functions, but the majority are classified as transcription cofactors, coactivators, and DNA-binding proteins, or regulators of metabolic processes (Butland et al., 2007). In mammalian cells, poly-Q expanded Htt globally disrupts transcriptional regulation (Steffan et al., 2000; Dunah et al., 2002; Schaffar et al., 2015). In a yeast two-hybrid assay, transcriptional activity of aggregation-prone Htt was dependent on the length of the poly-Q tract (Gerber et al., 1994; Benn et al., 2008; Atanesyan et al., 2012). These observations underscore the importance of understanding the functional role that Htt has in the nucleus.

We reasoned that Slx5, owing to it’s protein quality-control functions, may alter the aggregation or distribution of Htt aggregates in WT cells. Therefore, we investigated whether STUbLs, Slx5, and Slx8 play a role in preventing the toxicity of poly-Q expanded Htt in budding yeast cells. We found that expression of Htt-103Q elicited a severe growth defect and was toxic in *slx5*Δ and *slx8*Δ mutants. The genetic interaction of Htt with STUbLs led us to examine the functional role of STUbLs in counteracting the toxic effects of Htt-103Q. For this we assessed the interaction of STUbLs with various Htt constructs using a reporter gene assay. Using this assay, we established that both Slx5 and RNF4, the human STUbL ortholog, reduced the transcriptional activity of Htt in yeast and human cells. Functionally, we determined that a plasmid-borne copy of *SLX5* reduced the levels of both cytosolic and nuclear Htt aggregates but did not affect the levels of monomeric Htt protein in the nucleus. Finally, we completed a global RNA sequencing study to identify transcripts that are affected by Htt-103Q and modulated by an extra copy of *SLX5*. Therefore, our data implicates STUbLs in a conserved mechanism that prevents the accumulation of aggregating proteins such as Htt on chromatin and curbs their promiscuous transcriptional activity both in yeast and in mammalian cells.

## Materials and Methods

### Yeast Strains, Plasmids, Mammalian Tissue Culture, and Media

All strains and plasmids used in this study are listed in **Supplementary Table S1**. Unless noted otherwise, preparation of yeast media and manipulation of yeast strains were performed as previously reported (Guthrie and Fink, 2002). Unless otherwise noted, all yeast strains were grown at 30°C. Yeast plasmids expressing 25Q and 103Q Htt were purchased from Addgene.org (Addgene plasmid # 1177 (GPD-25Q-GFP Htt in p416), # 1180 (GPD-103Q-GFP Htt in p416)). These plasmids were used for growth assays and microscopy. For Htt localization and auto-activation assays, Htt with 25Q, 55Q, and 97Q were PCR-amplified using NEB Q5 hot start high-fidelity polymerase 2× master mix (Cat # M0494S) and cloned into the pCR8/GW/TOPO entry vector (Life Technologies) and then recombined into either pAG414GAL-ccdB-DsRed (Addgene #14359) forming GAL-97QHtt-DsRed/TRP1/CEN (BOK 1213) or pACT2.2gtwy (Addgene # 11346) forming ADH1-GAL4AD-25QHtt/LEU2/2μ (BOK 1207), ADH1-GAL4AD-55QHtt/LEU2/2μ (BOK1209) and ADH1-GAL4AD-97QHtt/LEU2/2μ (BOK 1215). All plasmids expressing Htt encode exon I (17 amino acids) followed by poly-Q and proline-rich regions. NEBase Changer v1.2.1 software at NEB website was used for designing mutagenesis 5′-phospho primers and NEB Q5 hot start high-fidelity polymerase 2x master mix was used for PCR amplification. After PCR, template plasmid DNA in the reaction mixture was digested by treatment with DpnI enzyme (cat # R0176S) and PCR amplicon was ligated using T4 DNA Ligase enzyme (Cat # M0202S). All primer sequences used for cloning and mutagenesis are available upon request. Yeast cells were transformed as previously described (Amberg et al., 2005) or using the frozen-EZ yeast transformation II kit (Zymo research corporation, Irvine CA). For mammalian 2-hybrid assays, the Matchmaker Mammalian Assay Kit 2 (Clontech.com Cat. No. 630305) was used as per suppliers instructions. The pVP16 Activation Domain (AD) Htt constructs were designed in the Kerscher lab and synthesized by Genewiz (South Plainville, NJ, United States) to produce pVP16-Htt25Q-AD and pVP16-Htt55Q-AD. RNF4 was PCR amplified and cloned into *EcoR*1 and *Hind*III sites in the pM-BD plasmid to produce pM-BD-RNF4.

PC3 (Prostate Adenocarcinoma), PNT2 (Prostate Epithelium), and LNCaP (Prostate Carcinoma) cells were grown in RPMI media with 10% heat inactivated FBS (Thermo Fisher Scientific #10438018) and 1% antifungal/antibiotic (anti/anti) (Thermo Fisher Scientific #15240062). PC12 (Rat pheochromocytoma) cells were grown as above but also contained 10% horse serum. HEK 293 (Embryonic Kidney) cells were grown in DMEM media with 10% heat inactivated FBS and 1% anti-anti. Cells were transfected using Lipofectamine 2000 or 3000 reagents using supplier instruction (Thermo Fisher Scientific, Cat. No. 11668-019 or L3000-015).

### Growth Curves

Yeast strains YOK2206-2207, YOK2209-2210, YOK2824-2828 were grown overnight in 5 ml selective media with 2% dextrose. OD readings were recorded every hour from OD_600_ ∼ 0.15 to OD_600_ ∼2.0 for up to 10 h (Thermo Fisher Scientific Spectronic 200). Readings were averaged and graphed in Microsoft Excel. Error bars represent the standard error of four independent cultures for each strain listed. Doubling times were calculated as previously published (Murakami and Kaeberlein, 2009).

### Spotting Assays

Yeast strains were grown overnight in 5 ml selective media with 2% dextrose. When cultures reached mid-log phase (OD_600_ 0.8–1.0), 1 OD of cells was harvested. Cultures were 10-fold serially diluted and 5 μl was spotted onto selective medium containing 2% dextrose. Plates were dried at ambient temperature and incubated at 30°C for up to 3 days.

### Ortho-Nitrophenyl-β-Galactoside (ONPG) and SEAP Assays

Yeast cultures of pJ694alpha containing the appropriate AD and BD constructs were grown until cells reached mid-log phase (OD_600_ of 1 ml = 0.5–0.8) and lac-Z reporter gene expression was determined as outlined in the Clontech Yeast Methods protocols handbook (PT3024-1). Briefly, the exact OD_600_ was recorded when the cultures were harvested. Cells were then washed in Z-buffer (16.1 g/L of Na_2_HPO_4_⋅7H_2_O, 5.50 g/L of NaH_2_PO_4_⋅H_2_O, 0.75 g/L of KCl, and 0.246 g/L of MgSO_4_⋅7H_2_O. pH 7.0). Cell pellets were resuspended in 100 μl of Z-buffer and three cycles of freeze/thaw each for 30 s was done to break open the cells. Cells were then incubated in the presence of ONPG (4 mg/ml) in Z-buffer at 30°C until yellow color developed. Reactions were stopped using 1 M Na_2_CO_3_ and cell debris was removed by centrifugation. The OD_420_ was determined using a spectrophotometer and β-galactosidase units were calculated using the formula [β-gal units = 1000 × OD_420_/(*t* × V × OD_600_)] where t is elapsed time (in minutes) of incubation, V is 0.1 ml times 5 (concentration factor) (Miller, 1972). The β-galactosidase units reported were average values of at least three independent experiments and values were graphed including +/−*SD*. The Great EscAPe Chemiluminescence kit (Clontech #631737) was used to detect SEAP levels in the mammalian 2-hybrid assay. Twenty-five microliters of culture media were obtained and spun for 1 min at 12,000 rpm to remove cells. The supernatant was transferred to black 96-well plates with clear, flat well bottoms (Corning #353219) and after addition of SEAP substrate solution, Chemiluminescent signals were visualized and analyzed using a Li-COR C-Digit Blot Scanner and also autoradiography film. Student’s *t*-tests were used to analyze statistical significance of SEAP transcriptional levels.

### Fluorescence Microscopy

Images of live cells were collected using a Zeiss Axioscope two plus microscope (Carl Zeiss Microscopy, LLC, Thornwood, NY, United States) fitted with a Qimaging Retiga^TM^ SRV charge-coupled device digital camera (Qimaging, Surrey, BC, Canada), i-Vision software for macintosh (Bio Vision Technologies, Exton, PA, United States) and a Uniblitz shutter assembly (Vincent Associates/ UNIBLITZ, Rochester, NY, United States). Pertinent filter sets for the above applications include CZ909 (GFP), XF114-2 (CFP), Filter set 15 (DsRed1), and 49 (DAPI and Hoechst 33258) (Chroma Technology Group, Bellows Falls, VT, United States). Where applicable, images were normalized using i-vision software and pseudo-colored and adjusted using Adobe Photoshop software (vs13.0 × 64, Adobe Systems, San Jose, CA, United States).

### Subcellular Fractionation Assay

Cells were grown in a 2% raffinose synthetic complete medium at 25°C until reaching mid-log phase. Then, galactose was added to the media to a final concentration of 2% to induce Htt-25Q-NLS-GFP or Htt-103Q-NLS-GFP expression from the *GAL* promoter for 4 h at 25°C. Whole cell extract (WCE) was purified from 50 OD_600_ equivalent cells. Subcellular fractionation was performed as described previously (Au et al., 2008). Western blot analysis of WCE, soluble, and chromatin fraction was carried out to monitor the Htt25Q-NLS or Htt103Q-NLS levels. Tub2 and histone H3 were used as markers for soluble and chromatin fractions, respectively. Protein levels were quantified using Gene Tools software (version 3.8.8.0) from SynGene (Frederick, MD, United States). Primary antibodies were anti-GFP mouse (1:3000, 11814460001, Roche), anti-Tub2 rabbit (1:3000, Basrai laboratory), and anti-H3 rabbit (1:7500, ab1791, Abcam).

### Total RNA Isolation

Cells were grown in a 2% raffinose synthetic complete medium at 25°C until reaching mid-log phase. Then, galactose was added to the media to a final concentration of 2% to induce Htt-25Q-NLS or Htt103Q-NLS expression from the *GAL* promoter for 4 h at 25°C. Total RNAs were isolated from 3 OD_600_ equivalent cells using MasterPure^TM^ Yeast RNA purification kit with DNase I treatment as indicated by the manufacturer (Epicentre). All RNA samples had an RNA integrity number (RIN) of 8 and above, indicative of high sample quality. Half of the sample is used for RNA sequencing, and another half is for RT-PCR for a validation of the RNA sequencing.

### Reverse Transcription-PCR (RT-PCR)

Total RNAs (100 ng for *HBT1*, 10 ng for *UIP4* and *UBC11*, and 1 ng for *ACT1*) were analyzed by AccessQuick^TM^ RT-PCR system (Promega). Primer sets and PCR conditions are available upon request. PCR products were loaded onto Ethidium Bromide-stained 1.5% agarose gels in TBE (KD Medical) and band intensities were quantified with Gene Tools software (version 3.8.8.0) from SynGene (Frederick, MD, United States). Expression levels were calculated based on the standard curve on the same gel and relative values were determined when level of the NLS-Htt25Q-GFP [Vector] was defined as 100.

### mRNA-Seq and Analysis

Three independent RNA-seq libraries for each of 4 samples were prepared from total RNA using the Illumina TruSeq Stranded Total RNA Kit RS-122-2201. They were pooled and sequenced in a single 150 cycle paired end HiSeq run at the Frederick National Laboratory for Cancer Research (FNLCR) at the CCR Sequencing Facility, NCI, NIH, Frederick, MD 21701. Fifty-six to 81 million pass-filter reads were obtained with > 95% base calling quality of Q30. Reads were adapter-trimmed with low-quality calls removed using Trimmomatic v0.36 and aligned using STAR 2.5.1. The transcriptome reference was annotated transcripts from *Saccharomyces cerevisiae* S288C, assembly EF4 (Ensembl). One library (YMB10544_c) contained 45% rRNA sequences and was removed from further analysis (all other libraries contained < 2% rRNA reads). Genewise read counts were quantitated using RSEM 1.2.22, and differential expression analysis was performed using edgeR version 3.20.9 utilizing the tool’s GLM functionality. An F-like test was performed first to identify genes showing a statistically significant difference in at least one condition (3961 of 7126 total), and only these genes were included in subsequent pairwise comparisons. Analysis of identified transcripts was completed using the online Panther classification system ^1^ (Mi et al., 2013) and the Saccharomyces Genome Database ^2^.

## Results

### Expression of Poly-Q Expanded Huntingtin Causes a Growth Defect in STUbL Mutants

**FIGURE 1:**
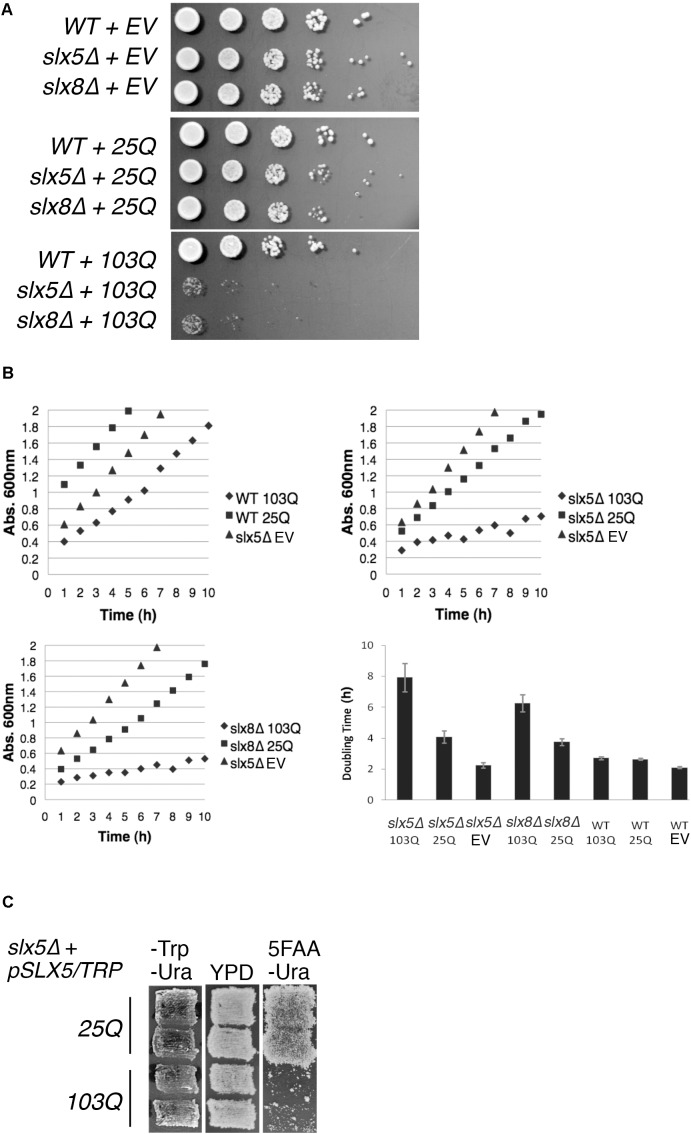
STUbL subunits Slx5 and Slx8 alleviate toxicity of poly-Q expanded Htt. **(A)** WT, *slx5*Δ, and *slx8*Δ strains expressing Htt-25Q, Htt-103Q, or empty vector (EV) were grown to mid-logarithmic phase and 5 μl of 10-fold serial dilutions of each culture were spotted on SC-URA medium. Plates were incubated at 30°C for 3 days. **(B)** Yeast transformants in **A** and the indicated controls were grown overnight in 5 ml of SC-URA medium. Ten OD_600_ readings of cultures were recorded every hour until the OD_600_ reached ∼2.0. The average doubling times of four independent experiments were graphed with +/− standard error. EV (empty vector) **(C)** A shuffle strain, *slx5*Δ with *SLX5/TRP* plasmid (YOK 2990), was transformed with either Htt-25Q or Htt-103Q constructs. Transformants were patched in duplicate on selective medium (SC-TRP URA) and rich medium (YPD). Patches were then replica plated on SC-URA medium with 5FAA to counter-select against the *TRP1* marked plasmid.

STUbLs play an important role in the quality control of both SUMO-modified and non-sumoylated proteins (Wang et al., 2006; Xie et al., 2010; Westerbeck et al., 2014). Therefore, we tested our hypothesis that Slx5 and Slx8 are required for growth in the presence of a toxic, aggregation-prone model protein: exon 1 of poly-Q expanded Htt. Budding yeast is established as an exquisite model system for the study of poly-Q expanded proteins (Krobitsch and Lindquist, 2000) and hence we compared the effect of expression of Htt with either a 25-glutamine residue tract (Htt-25Q or 25Q) or an abnormal, aggregation-prone 103-glutamine tract (Htt-103Q or 103Q) on the growth properties of WT, *slx5*Δ, and *slx8*Δ cells. Isogenic WT, *slx5*Δ, and *slx8*Δ cells were transformed with low-copy (*CEN*) plasmids expressing GFP-tagged Htt-25Q or Htt-103Q under control of the constitutive GPD promoter (Krobitsch and Lindquist, 2000). The resulting transformants, or an empty vector control, were grown to mid-log phase and equal numbers of cells were serially diluted and spotted on selective media (**Figure 1A**). Though *slx5*Δ and *slx8*Δ cells initially formed smaller colonies than WT cells, no severe growth defect or lethality was apparent after 2–3 days of growth at 30°C for both the vector and Htt-25Q transformants (**Figure 1A** top and middle panel). In contrast, *slx5*Δ and *slx8*Δ cells transformed with the Htt-103Q construct showed a severe growth defect (**Figure 1A** bottom panel), supporting our hypothesis that STUbLs are required to relieve the growth-inhibiting properties of aggregation-prone poly-Q expanded Htt in budding yeast.

Next, we used liquid cultures to investigate the effect of Htt-25Q and Htt-103Q constructs on the growth of WT, *slx5*Δ, and *slx8*Δ. For this analysis, we compared the slopes of growth curves for WT, *slx5*Δ, and *slx8*Δ cells that were transformed with either Htt-25Q or Htt-103Q constructs. First, we found that WT cells transformed with Htt-25Q or Htt-103Q grew equally well as *slx5*Δ cells once established in logarithmic phase of growth (**Figure 1B** top left). Similarly, *slx5*Δ and *slx8*Δ cells transformed with either an empty vector or the Htt-25Q construct displayed similar growth characteristics (**Figure 1B**, top right and bottom left). In sharp contrast, the growth curves for *slx5*Δ and *slx8*Δ cells transformed with 103Q constructs revealed a significant growth delay with two- to four-fold increases in doubling times from 2 to 8 h (**Figure 1B** bottom right).

Further support that STUbL subunits, Slx5, and Slx8, have a role in preventing the Htt-103Q induced growth delay or toxicity was derived from a shuffle assay used to examine the ability of a *slx5*Δ STUbL mutant to grow in the presence of Htt-25Q or Htt-103Q. For this assay, a *slx5*Δ shuffle strain (*slx5*Δ; *SLX5/TRP1/CEN*) was transformed with Htt-25Q or Htt-103Q. All transformants showed similar growth characteristics and were patched in duplicate onto selective (-TRP -URA) media. Once patches grew in, cells were replica-plated on rich media (YPD) and then onto 5FAA media to counter-select against the *TRP*-marked WT *SLX5* plasmid (Toyn et al., 2000). After two successive replicas onto fresh 5FAA media, the majority of cells with the Htt-103Q construct failed to grow into colonies because they had lost *SLX5*. In stark contrast, *slx5*Δ cells harboring Htt-25Q grew unimpeded because growth of these cells did not depend on *SLX5* (**Figure 1C**). In summary, our results show that STUbLs provide an essential function for yeast cells growing in the presence of aggregation-prone, poly-Q expanded proteins.

### Slx5 Reduces the Number of Poly-Q Expanded Huntingtin Aggregates

Intrigued by the poly-Q-induced growth defect in both STUbL mutants, we decided to compare the phenotypic manifestations of aggregation-prone Htt in WT, *slx5*Δ, and *slx8*Δ cells. We used a fluorescence microscope to collect images of WT, *slx5*Δ, and *slx8*Δ cells transformed with either the GFP-tagged 25Q construct or a GFP-tagged 103Q construct. We predicted, based on the results of our growth assays (**Figure 1**), that STUbL mutants would affect the localization of 103Q construct but not the 25Q construct. Consistent with previous results (Krobitsch and Lindquist, 2000), the Htt-25Q-GFP construct was evenly distributed across the nucleus and cytosol of WT, *slx5*Δ and *slx8*Δ cells (data not shown). In contrast, WT cells expressing Htt-103Q-GFP revealed a mixture of speckles, aggregates, and diffuse-staining cells (see **Supplementary Figure S1**). However, the majority of *slx5*Δ and *slx8*Δ cells expressing 103Q did not reveal a GFP signal as these cells were dead, as determined by a vital stain that differentiates live and dead cells (**Supplementary Figure S1**). By comparison, WT cells expressing 103Q contained less than 5% of dead or dying cells in the culture. These results also show that constitutive expression of 103Q, unlike 25Q, results in lethality of STUbL mutants. These data are consistent with our growth assays (**Figures 1A–C**) and support our conclusion that STUbLs fulfill an essential role in preventing cytotoxicity due to poly-Q expanded proteins such as Htt-103Q.

**FIGURE 2:**
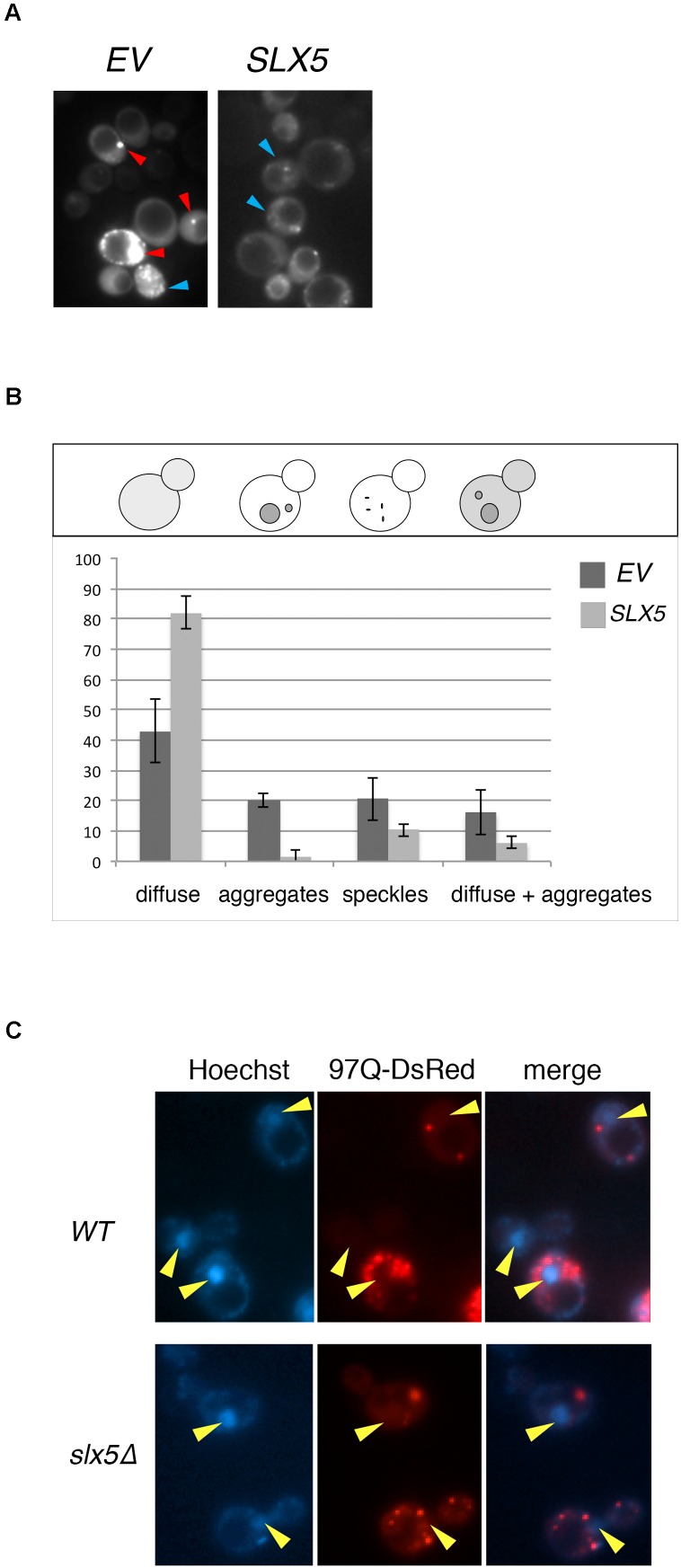
Plasmid-borne SLX5 reduces aggregates of poly-Q expanded huntingtin. **(A)** Representative images of Htt-103Q in WT cells with and without an *SLX5/CEN* plasmid. Example of aggregates (clearly defined, bright cytoplasmic structures – red arrow-heads) and speckles (multiple small, not clearly defined cytoplasmic granules – blue arrow-heads) **(B)** Quantitation of phenotypes observed in 2A. WT strain expressing Htt-103Q-GFP alone (YOK 2842) or Htt-103Q-GFP with *SLX5* (YOK 2843) were grown to mid-logarithmic phase in selective medium. Images of 103Q diffuse staining, aggregates, and speckles in the indicated strains were recorded and then quantitated. Average counts for three independent experiments were graphed +/− standard deviation. Y-axis: percent of cells (*n* = 100/experiment). Y-axis: phenotypes scored **(C)** Aggregates of poly-Q expanded Htt are localized in the cytoplasm. WT and *slx5*Δ strains transformed with GAL-Htt-97Q-DsRed (YOK 3112 and YOK 3114) were grown overnight in SC-TRP medium with 2% raffinose. Cultures were diluted to ∼0.2 OD in a fresh medium with 2% galactose and incubated for an additional 16 h for expression of Htt-97Q-DsRed prior to imaging Htt aggregates using a fluorescence microscope. Nuclei were stained with Hoechst dye. Merged images indicate the absence of Htt-97Q aggregates in nuclei (yellow arrow-heads).

The Htt-103Q-induced lethality in *slx5*Δ strain impeded our microscopic analysis of Htt toxicity in STUbL mutants and hence we assayed the effect of plasmid-borne *SLX5* on the phenotype of 103Q aggregates in WT cells. We reasoned that Slx5, owing to it’s quality-control functions, may alter the aggregation or distribution of Htt aggregates in WT cells. WT cells were transformed with GFP-tagged 103Q and either a *SLX5* *CEN* plasmid (under control of its own promoter on a low-copy *CEN* vector) or an empty control vector. The transformants were grown to mid-logarithmic phase in selective media and 103Q aggregates were analyzed using fluorescence microscopy (**Figure 2A**). We determined that the incidence of 103Q aggregates was reduced by almost 14-fold while the number of diffuse-staining cells increased by at least twofold with plasmid borne *SLX5* (**Figure 2B**).

Htt has been reported to reside both in the cytosol as well as the nucleus, but the majority of Htt aggregates are observed to form in the cytosol (Davies et al., 1997; Krobitsch and Lindquist, 2000). The nuclear localization of Slx5 and Slx8 (Cook et al., 2009) and the lethality of Htt103Q in *slx5*Δ and *slx8*Δ strains prompted us to further investigate the role of STUbLs in the localization of Htt. For this analysis, we transiently expressed 97Q-DsRed under control of the strong inducible *GAL promoter* in WT and the *slx5*Δ mutant. This transient expression prevented the cytotoxicity associated with constitutive expression of aggregation-prone Htt in *slx5*Δ strains. After galactose induction, we imaged the nuclei of live WT and *slx5*Δ cells were stained with Hoechst dye (33342). In both WT and *slx5*Δ cells (*n* > 200), Htt aggregates or speckles were solely observed in the cytosol. A low level of diffusely staining 97Q-DsRed was evenly distributed between the cytosol and the nucleus of WT and did not appear to be enriched in either compartment. A similar localization pattern for 97Q-DsRed to that in WT cells was observed in the *slx5*Δ cells (**Figure 2C**). Based on these results we propose that 97Q, under the conditions employed, does not readily form large aggregates in the nuclei of yeast cells.

### Regulation of Transcriptional Activity of Htt by Slx5 and RNF4

**FIGURE 3:**
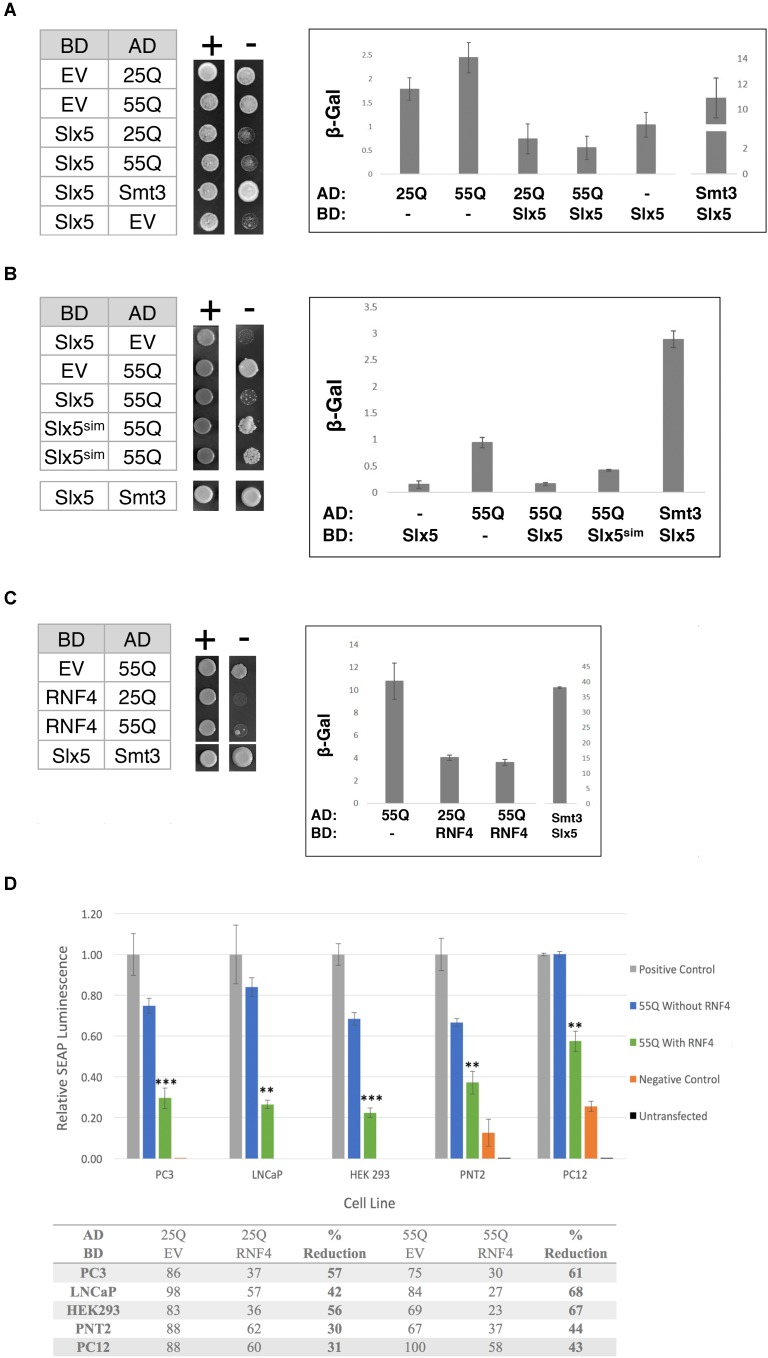
The STUbL subunit Slx5 reduces the transcriptional auto-activation of poly-Q expanded Htt. **(A)** Yeast two-hybrid strain pJ694α was co-transformed with the indicated AD and BD plasmids. 1 OD of overnight-grown cells were diluted 100-fold and 5 μl of each cell suspension was spotted on SC-TRP LEU for growth control and SC-TRP LEU HIS to assess activation of the *HIS3* reporter gene. Transcriptional activation was also scored by quantification of β-galactosidase activity using ONPG assays (error bar – *SD*). **(B)** Effect of Slx5^sim^ expression on the repression of poly-Q expanded Htt auto-activation as indicated by growth on SC-TRP LEU HIS medium and β-galactosidase assays. **(C)** Expression of the human *SLX5* ortholog RNF4 represses the transcriptional activity of poly-Q expanded Htt. To assess activation of the *lacZ* reporter gene in the indicated strains β-galactosidase units of strains were determined and graphed +/− standard deviation. **(D)** RNF4 significantly reduces the transcriptional activation of Htt in mammalian cells. Mammalian two hybrid analysis of PC3, LNCaP, HEK 293, PNT2, and PC12 cells [*n* = 3]. Reporter gene auto-activation of 25Q and 55Q Htt was assessed by transfection of pM-EV (empty vector) and pVP16-Htt. Where indicated, pM-RNF4 was cotransfected with 25Q or 55Q Htt to measure RNF4’s inhibitory function. Values of relative SEAP luminescence for 55Q are graphed as shown and values for both 25Q and 55Q are shown in the table below the graph. Assay results were normalized to the positive control. Error bars represent standard deviation. ^∗∗^*p* < 0.01, ^∗∗∗^*p* < 0.001.

It was previously reported that poly-Q expanded Htt, in the absence of a Gal4-DNA-binding fusion (BD), induces the expression of reporter genes in a two-hybrid reporter assay (Atanesyan et al., 2012). This transcriptional auto-activation was directly related to the length of the poly-glutamine tract in Htt (Atanesyan et al., 2012). Therefore, we determined the effect of Slx5 on this poly-Q dependent transcriptional activity. Htt-25Q and Htt-55Q were fused to the Gal4 activation domain (AD) and assayed for the auto-activation of each construct in the presence or absence of BD-Slx5 or just BD. Consistent with published data (Atanesyan et al., 2012), all AD fusions of Htt, by themselves, induced expression of both a *HIS3* and a *lacZ* reporter gene, indicating that both 25Q and 55Q associate with the Gal4-UAS independent of a BD (**Figure 3A**). We used AD-Htt-25Q and AD-Htt-55Q to avoid the potential toxicity associated with AD-Htt-97Q. Auto-activation of the *HIS3* reporter gene was scored using a growth assay, transformed cells where diluted and spotted on media with (*SD*-Trp-Leu) or without histidine (*SD*-Trp-Leu-His). Concomitantly, auto-activation of the *lacZ* reporter was quantitated using ONPG assays that were performed in triplicate. Intriguingly, when the AD-Htt constructs were paired with BD-Slx5, the Htt-induced auto-activation was reduced to background levels (**Figure 3A**). To confirm that the reduction of the Htt-induced transcriptional auto-activation is not due to the decreased expression of Htt 25Q and Htt 55Q in the presence of *BD-SLX5*, we tested the expression of AD-Htt 25Q and AD-Htt-55Q by Western blotting (**Supplementary Figure S3**). The result shows that the steady state levels of both Htt-25Q and Htt-55Q were not affected by *BD-SLX5*.

Next we asked if the suppression of AD-Htt reporter gene activation was dependent on the SIMs in Slx5. We combined a SIM mutant of Slx5 that fails to interact with SUMO (BD-Slx5^sim^) with AD-55Q and assessed the auto-activation properties of our poly-Q Htt constructs on the two reporters using growth and quantitative ONPG assays. We found that Slx5^sim^ reduced the auto-activation of the AD-55Q construct significantly less than WT Slx5 (**Figure 3B**, ONPG assay). This data suggests that SUMO-binding may support the ability of Slx5 to suppress the transcriptional activity of AD-Htt. However, SUMO-binding of Slx5 may not be a steadfast requirement to reduce Htt toxicity because the Slx5^sim^ mutant can still suppress the Htt-103Q growth phenotype (**Supplementary Figure S2**).

To determine whether ability of Slx5 to repress auto-activation of poly-Q expanded Htt is evolutionarily conserved, we tested mammalian BD-RNF4 in combination with AD-25Q and AD-55Q. Consistent with results for Slx5, RNF4 also repressed the auto-activation activity of AD-25Q and AD-55Q constructs in our reporter assay (**Figure 3C**). In the presence of RNF4, the auto-activation activity of AD-25Q and AD-55Q was reduced threefold, when compared to AD-55Q alone. These results strongly support a role for RNF4, and other STUbLs, in counteracting the aggregation of transcriptionally active Htt and possibly other poly-Q expanded proteins associated with neurodegenerative diseases.

Finally, we tested whether RNF4 curbs the transcriptional activity of aggregation-prone proteins in a mammalian tissue culture model of Huntington’s disease, employing a mammalian Matchmaker (2-hybrid) assay. For this approach, both Htt-25Q and Htt-55Q were cloned into the pVP16AD Gal4-activation domain vector and co-transformed with the reporter plasmid pG5SEAP into 5 separate cell lines (PC3, LNCaP, HEK293, PNT2, and PC12). Consistent with our finding in yeast, all mammalian cell lines recapitulated the Htt-dependent transcription of the pG5SEAP reporter (**Figure 3D** – blue bars). Next, reporter gene activation was assayed in the presence of BD-RNF4. Mammalian two-hybrid analysis in all cell lines displayed a significant decrease in the transcriptional activation of 25Q and 55Q Htt upon addition of RNF4 BD (**Figure 3D** green bars and table). The strength of RNF4’s inhibitory effect ranged from 30–60% reduction on 25Q but was statistically significant for all 5 cell lines (Student’s *T*-test), indicating that RNF4’s inhibitory effect is consistent and reproducible. In all cell lines, 55Q mHtt displayed greater transcriptional reduction (40–70%), suggesting that this poly-Q expanded 55Q Htt is more amenable to RNF4’s activity. Due to their neuronal origin, results from the PC12 cell two-hybrid are the most physiologically relevant model of Huntington’s Disease. As an important indicator of specificity, reporter gene activation by a positive control construct (pM-pVP16), was not affected by transfection of RNF4 (data not shown). In summary, we have now shown that both in yeast and mammalian cells auto-activation of Htt can be significantly modulated due to the activity of STUbLs.

### Slx5 Reduces Chromatin-Associated Htt Aggregates in Budding Yeast

**FIGURE 4:**
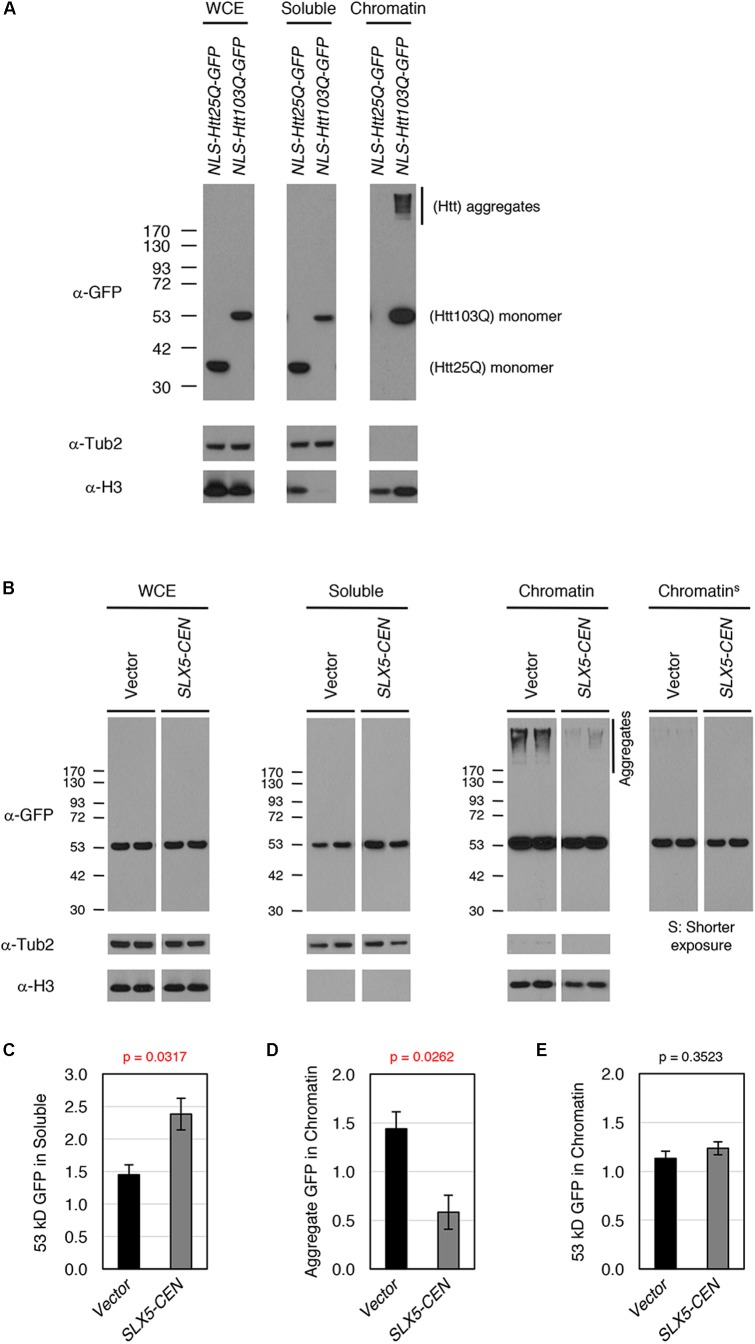
Slx5 reduces the level of chromatin associated Htt103Q aggregates. **(A)** Htt103Q, but not Htt25Q, associates with chromatin. Whole cell extracts (WCEs) prepared from equal numbers of cells expressing GFP-tagged Htt25Q-NLS or Htt103Q-NLS from a *GAL* promoter were fractionated into soluble and chromatin fractions. Htt25Q-NLS-GFP or Htt103Q-NLS-GFP levels in each fraction were monitored by western blot analysis with anti-GFP antibody. Tub2 and histone H3 were used as markers for soluble and chromatin fractions, respectively. **(B)** WCEs prepared from equal numbers of cells expressing Htt103Q-NLS-GFP with (*SLX5-CEN*) or without (Vector) were fractionated into soluble and chromatin fractions as described in **A**. Htt103Q-NLS-GFP levels were monitored by western blot analysis with anti-GFP antibody. Three independent transformants were assayed and shown are the results from two of these. **(C)** Quantification of the 53 kD GFP signals in soluble fraction from 4B. The 53 kD GFP was normalized using Tub2 levels in soluble fraction. The graph represents the mean of three independent clones with SEM. *P*-value is 0.0317. **(D)** Quantification of the aggregate GFP signals in chromatin fraction from 4B. The aggregate GFP signal was normalized using H3 levels in chromatin fraction. The graph represents the mean of three independent transformants with SEM. *P*-value is 0.0262. **(E)** Quantification of the 53 kD GFP signals in chromatin fraction from 4B. The 53 kD GFP (shorter exposure) was normalized using H3 levels in chromatin fraction. The graph represents the mean of three independent transformants with SEM. *P*-value is 0.3523.

To study the physiological relevance of our assays for transcriptional activity, we examined whether Htt-103Q associates with chromatin using subcellular fractionation of whole-cell lysates after overexpression of nuclear targeted Htt-25Q-NLS-GFP or Htt-103Q-NLS-GFP. We assayed levels of Htt in whole-cell extracts, soluble fractions, and chromatin (**Figure 4A**). As expected, both aggregated (high-molecular weight) and non-aggregated (53 kD, monomer) forms of Htt-103Q, but not Htt-25Q, were clearly detectable in the chromatin fraction, indicating that both aggregated and non-aggregated Htt-103Q associate with chromatin.

To examine the role of *SLX5* in modulating chromatin bound Htt-103Q, we assayed levels of Htt-103Q-NLS-GFP in the presence or absence of plasmid-borne *SLX5* (**Figures 4B–E**). Consistent with the microscopy of Htt-103Q-GFP expressing cells (**Figure 2B**), the soluble, monomeric form of Htt-103Q (53 kDa) was increased with plasmid-borne *SLX5* (**Figures 4B,C**). Importantly, aggregated Htt in the chromatin fraction was reduced ∼3-fold (*p* = 0.0262) in the strain with plasmid borne *SLX5* (**Figures 4B,D**). In contrast, monomeric, chromatin-bound Htt-103Q (53 kDa) remained similar in both strains (**Figures 4B,E**). These data show that increased expression of *SLX5* specifically reduces Htt-103Q aggregates in chromatin. We propose that STUbLs contribute to reducing chromatin-associated Htt aggregates.

### Identification of Htt-Altered Transcripts Modulated by a STUbL in Yeast

**FIGURE 5:**
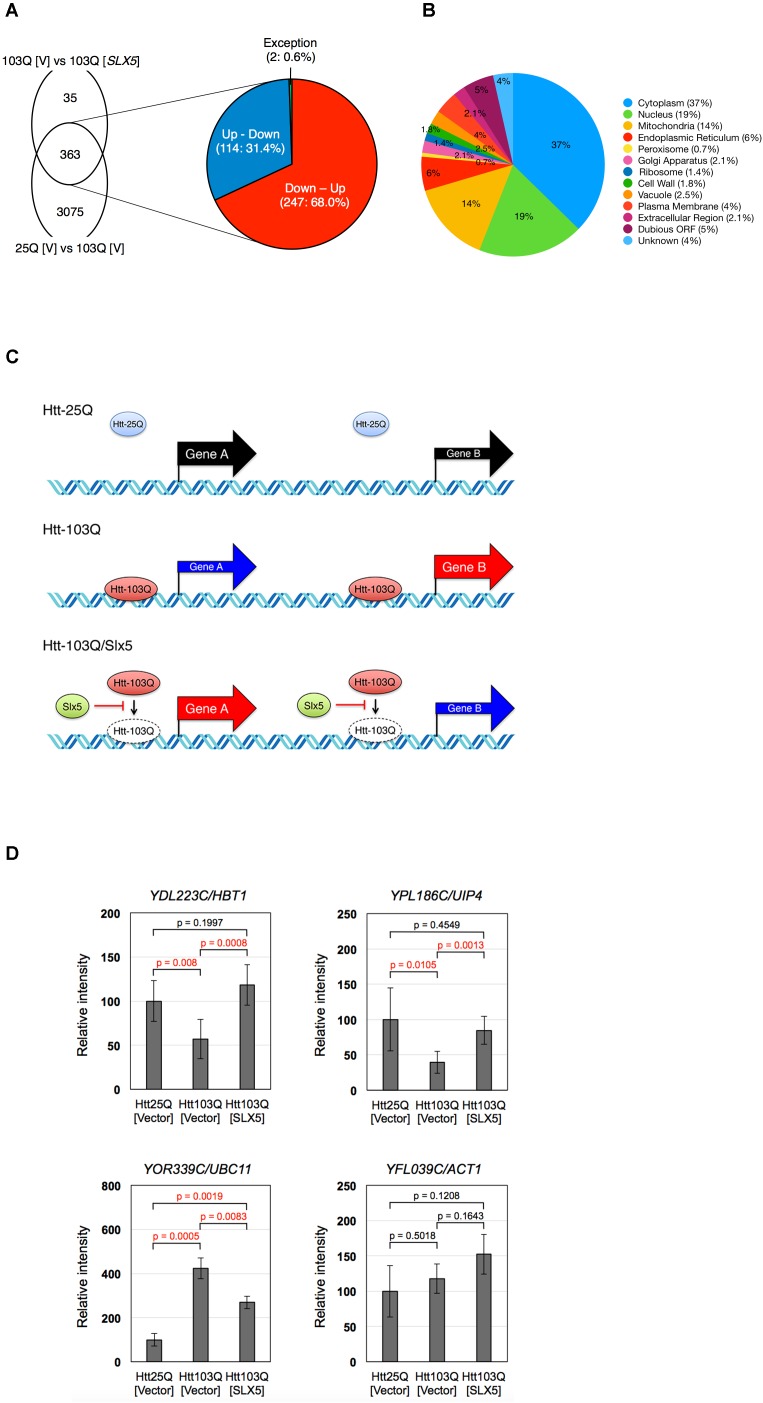
Slx5 modulates the transcriptional activity due to expression of Htt-103Q-NLS. Depiction of 398 *SLX5* modulated genes identified by RNA sequencing. **(A)** RNA-seq analysis shows that Htt103Q-NLS leads to a global effect on the transcriptome as it affects the expression of 3438 genes (25Q [V] vs. 103Q [V]). Plasmid-borne *SLX5* affects the expression of 398 genes in Htt103Q-NLS cells (103Q [V] vs. 103Q [*SLX5*]) as shown in **Supplementary Table S2**. The overlap between Htt25Q-NLS and Htt103Q-NLS (25Q [V] vs. 103Q [V]) is 363 genes. The expanded view of **A** shows that expression of most of the 363 genes (99.4%) inversely correlates between 25Q [V] vs. 103Q [V] and 103Q [V] vs. 103Q [*SLX5*]. Expression of 247 of the 363 genes (68.0%) is down-regulated by Htt103Q, and this effect is reversed by plasmid-borne *SLX5* (Down – Up). Expression of 114 of the 363 genes (31.4%) is up-regulated by the Htt103Q, and this effect is reversed by plasmid-borne *SLX5* (Up – Down). **(B)** Subcellular localization of differentially expressed genes. The localization of proteins encoded by the 398 genes indicated in **Supplementary Table S2** was analyzed using cellular components assignment from the PANTHER Classification System and the Saccharomyces Genome Database. Pie chart shows a ratio of the genes placed into cellular component categories. Individual genes are listed in **Supplementary Table S4**. **(C)** Schematic of gene expression in Htt25Q-NLS and Htt103Q-NLS cells and the effect of plasmid-borne *SLX5* on the transcriptome of Htt103Q-NLS cells. Expression of genes A and B are downregulated and upregulated in Htt103Q-NLS cells relative to Htt25Q-NLS cells, respectively. Slx5 reduces the association of Htt with chromatin and this contributes to the reversal in gene expression such that gene A is upregulated and gene B is downregulated. **(D)** RT-PCR validation of gene expression analysis. Total RNAs were purified from strains expressing either Htt25Q-NLS or Htt103Q-NLS from a *GAL* promoter for 4 h with or without plasmid-borne *SLX5*. RT-PCR analyses was performed using the same samples used for the RNA-seq. Relative intensities are reported as the mean ±*SD* of three biological repeats. Reactions for *YDL223C/HBT1* and *YPL186C/UIP4* were performed in duplicate. *N* = 3 for *YOR339C/UBC11* and *YFL039C/ACT1*, *N* = 6 for *YDL223C/HBT1* and *YPL186C/UIP4*.

The reduced association of aggregated Htt-103Q-NLS with chromatin in the presence of plasmid-borne *SLX5* (**Figure 4B**) led us to postulate that Slx5 curbs the abnormal transcriptional activities induced by Htt-103Q-NLS. Hence, we performed genome-wide RNA-seq analysis to examine the transcriptome of four strains expressing either Htt-25Q-NLS or Htt-103Q-NLS with or without plasmid-borne *SLX5*. Consistent with the effect of Htt-103Q on transcription, our results showed that the expression of > 50% of all yeast genes (3438 genes) was altered in the presence of Htt-103Q-NLS when compared to Htt-25Q-NLS with empty vector (**Figure 5A**, 25Q [V] vs. 103Q [V]). Of the 3438 genes affected by Htt-103Q-NLS, 48.6% of the genes were up-regulated and 51.4% were down-regulated. These results show that chromatin associated Htt-103Q-NLS affects global transcription in budding yeast.

We next analyzed the effect of plasmid-borne *SLX5* on the transcriptome of cells expressing Htt-25Q-NLS or Htt-103Q-NLS. Our RNA-seq data showed that *SLX5* had a minimal effect on the transcriptome of cells expressing Htt-25Q-NLS as only 33 genes were differentially expressed (25Q [V] vs. 25Q [*SLX5*]). In contrast to this, plasmid-borne *SLX5* affected the transcription of 398 genes in cells expressing Htt-103Q-NLS when compared to Htt-103Q-NLS without *SLX5* (**Figure 5A**, 103Q [V] vs. 103Q [*SLX5*]). The majority of the 398 genes encode for proteins that reside in the cytoplasm (37%), nucleus (19%), or mitochondria (14%) (**Figure 5B** and **Supplementary Table S4**). Of the 398 genes that were significantly altered by plasmid-borne *SLX5*, 66% (261) genes were upregulated and 34% (137) genes were down-regulated (**Supplementary Table S2**). We observed two distinctive characteristics on the transcriptome of Htt-103Q-NLS with and without plasmid-borne *SLX5* (**Figure 5C**). First, for 99.4% (361 out of 363) of *SLX5*-affected genes, the effect of added Slx5 was inversely correlated with that of 103Q (**Supplementary Table S2** and **Figure 5A**). Plasmid-borne *SLX5* upregulated the expression of 68.0% (247) of the genes that were downregulated by Htt-103Q-NLS, and downregulated the expression of 31.4% (114) of genes that were upregulated by Htt-103Q-NLS (**Figure 5A**). For example, expression of YAL008W was down-regulated by Htt-103Q-NLS, and up-regulated by plasmid-borne *SLX5*. In contrast, expression of YAL014C was up-regulated by Htt-103Q-NLS, and down-regulated by plasmid-borne *SLX5*. Only two genes (YDL182W and YGR092W) were an exception to this pattern (**Supplementary Table S2**). A second distinctive characteristic of the transcription profiles show that about 25% of the genes that are affected by plasmid-borne *SLX5* are neighbors or adjacent to each other on the chromosome (e.g., YBR052C, YBR053C, and YBR054W) (**Supplementary Table S3**). The RNA sequencing data generated in this study have been deposited in NCBI’s Gene Expression Omnibus (Barrett et al., 2013) and are accessible through GEO Series accession number GSE115990.

To confirm the transcriptome data from RNA-seq, we performed RT-PCR to assay the transcription of a subset of Htt-103Q-NLS/Slx5 modulated genes such as *YDL223C/HBT1, YPL186C/UIP4*, and *YOR339C/UBC11* (**Figure 5D**). Consistent with our RNA-seq data, we found that expression of *YDL223C/HBT1* and *YPL186C/UIP4* is down-regulated by Htt-103Q-NLS, and up-regulated by plasmid-borne *SLX5* (Down-Up). In contrast, *YOR339C/UBC11* is up-regulated by Htt-103Q-NLS, and down-regulated by plasmid-borne *SLX5* (Up-Down). In agreement with the RNA-seq data the expression of *ACT1* was not significantly affected when assayed by RT-PCR. In summary, our data shows that chromatin-associated Htt-103Q-NLS affects global transcriptional in budding yeast. Most importantly, we define a role for Slx5 in modulating the aberrant transcriptional activity, induced by chromatin-associated Htt-103Q-NLS.

## Discussion

In this study we show, for the first time, that STUbLs are required to prevent the toxicity associated with an aggregation-prone protein namely poly-Q expanded Htt and define a functional role for STUbLs in counteracting the toxic effects of Htt-103Q expression. Using reporter gene assays we determined that Slx5 and RNF4 reduce the transcriptional activity of Htt in yeast and human cells, respectively. For example, Htt fused to the Gal4 activation domain (AD) auto-activates Gal4-regulated reporter genes. However, reporter gene activation by Htt-AD is reduced to background levels in the presence of BD-Slx5 or BD-RNF4. Most importantly, our results show that Slx5 reduces cytosolic and chromatin-associated Htt-103Q aggregates and modulates the transcriptome of cells expressing Htt-103Q. Taken together we provide evidence for a conserved role of STUbLs in preventing the accumulation of aggregating proteins such as Htt on chromatin and propose that STUbLs counteract the transcriptional effect of these aggregates in yeast and mammalian cells.

In the initial stages of our analysis of Htt in STUbL mutants, we focused on the cellular distribution and aggregates formed by aggregation-prone Htt in yeast. We detected aggregates, speckles, and diffuse-staining Htt in both WT and a yeast STUbL mutant, *slx5*Δ (**Supplementary Figure S1**). Due to its toxicity in STUbL mutants, we ultimately studied the effect of an extra plasmid-borne copy of *SLX5* in WT cells expressing Htt-103Q. Presence of the *SLX5* plasmids increased diffusely staining Htt in WT cells while reducing the incidence of Htt aggregates (**Figures 2A,B**). However, we failed to detect a reproducible, STUbL-dependent, reduction of Htt by western blot analysis (for example **Supplementary Figure S3**). Previously it has been reported that the STUbL RNF4 is involved in the degradation of another poly-Q expanded protein, Atxn1 82Q (Guo et al., 2014). While our data are consistent with a re-distribution of Htt aggregate, we did not observe that Slx5 altered the steady-state levels of this aggregation-prone protein. One explanation for this may be that budding yeast cells do not disassemble the nuclear envelope, making it difficult to observe the effect of nuclear localized STUbLs on aggregates of Htt in the cytosol. We overcame this limitation in our yeast model by using nuclear-targeted Htt-103Q to assess both the transcriptional activity and the chromatin association of Htt in the presence or absence of *SLX5* (**Figure 3** and **Figure 4**).

First, using Gal4-based two-hybrid reporter assays, we were able to show that yeast Slx5 and human RNF4, both nuclear localized proteins, curb the transcriptional activity of Htt. Therefore, we predict that the role of RNF4 in mammalian cells is to dispel transcriptionally active Htt complexes rather than to degrade cytosolic Htt aggregates. However, at this point we cannot entirely exclude the possibility that Slx5 and RNF4 form repressive promotor-associated complexes see (Cubeñas-Potts and Matunis, 2013). Regarding the Gal4-AD-fusion of Htt, similar constructs have proven invaluable in defining the aberrant transcriptional activity of poly-Q expanded proteins. The most important observation in this regard is that the poly-Q domain is necessary and sufficient for both the targeting to the Gal4 UAS and reporter gene activation (Atanesyan et al., 2012). Furthermore, introduction of a poly-Q stretch into transcription factors increases their transcriptional activity. Even though we have not yet observed a direct, physical interaction between Htt and Slx5 (we predict a transient interaction involving Htt-associated proteins), our assays are consistent with an important role of Slx5 in counteracting nuclear activities of aggregation prone, chromatin associated proteins. The finding that a STUbL plays an important role in transcriptional regulation is not entirely surprising. Before it became known as a STUbL, RNF4 had already been identified as a co-regulator of androgen receptor-dependent transcription (Yan et al., 2002). Furthermore, RNF4 can act both as a transcriptional activator or a repressor depending on the proteins it interacts with (Fedele et al., 2000).

Second, using Gal-driven, nuclear-targeted 103Q constructs, we assessed the chromatin association of an aggregation-prone Htt construct. This time we were able to clearly document that an extra plasmid-borne copy of *SLX5* reduced the levels of chromatin-associated Htt (**Figure 4**). The association of Htt with DNA, transcription factor recognition elements, and transcription factors has previously been reported (Benn et al., 2008). STUbLs may provide a mechanism to counteract these inappropriate associations of Htt. For example, it is tempting to speculate that Slx5 recruits Cdc48/Ufd1/Npl4 (Cdc48-UN), a SUMO-targeted STUbL effector, to dislodge Htt from chromatin (Nie et al., 2012; Bergink et al., 2013). Cdc48 has also been identified in association with Htt aggregates and we are now studying the effect that Cdc48-UN plays in Htt-mediated transcriptional activation (Wang et al., 2008).

Finally, we have completed a global RNA sequencing study to identify those transcripts that are affected by nuclear-targeted Htt-103Q and modulated by an extra plasmid-borne copy of *SLX5*. Our transcriptome analysis revealed that *SLX5* counteracts transcriptional abnormalities of 398 genes induced by expression of 103Q-NLS. Dysregulated transcripts encode proteins localized throughout the cells, with the majority enriched in the cytoplasm (263) nucleus (33) and mitochondria (19). Functional categorization of the differentially transcribed genes showed that at least 22 are involved in transcription, transcriptional regulation, and RNA/DNA binding (*RTC3, RPA43, RPB7, BUD27, TFA2, SRB7, YAP7, MCM1, PHO4, MAP1, HSP31, SNF5, RPP1, TMA22, RPS27A, MRPL49, NHP2, RPL7A, RPL7B, MAP1, HST2*, and *CBC2*) (**Supplementary Table S4**). We posit that some of the transcriptionally active and chromatin-associated proteins identified in our study represent genuine STUbL targets. Additionally, several *SLX5*-modulated genes identified here have previously been described in other Htt studies [e.g., Glo2 (human HAGH1), ZTA1 (human zeta crystalline), Msb1, COA2, BUD22, ERG5, and TIR1], supporting a genuine role of STUbLs in counteracting huntingtin-mediated dysregulation (Willingham et al., 2003; Wolfe et al., 2014). We prefer a model in which Htt aggregates may contain both sumoylated and non-sumoylated proteins, including those listed above. STUbL-mediated ubiquitination could then result in the recruitment of the Cdc48-UN desegregase and the subsequent proteasomal degradation of ubiquitylated proteins in the aggregates (reviewed in Kerscher, 2016).

In summary, the STUbL/Htt assay is one of the first of its kind to assess the ability of RNF4 and other STUbLs to modulate the activity of transcriptionally active, aggregation-prone proteins. This reporter assay should complement other sophisticated genetic tools used to study protein aggregation processes (Newby et al., 2017). Results from our reporter assays are consistent with biochemical and genome-wide transcriptome data and provide evidence for a role of STUbLs in preventing toxicity due to aggregation-prone Htt in the nucleus. Overall, our findings indicate that STUbLs can reduce the chromatin association and abnormal transcriptionally activity of Htt (or other aggregating proteins) and suggest that mammalian STUbLs may play neuroprotective functions in Huntington’s Disease.

## Author Contributions

OK designed the study, drafted, wrote, and revised the manuscript, designed and completed the experiments, collected and interpreted the data, supervised the students and postdoc co-authors, approved the content for publication, and is accountable for all aspects of the work. KO, NP, and JP acquired, analyzed, and interpreted the data for the work, and revised the manuscript. GH analyzed and interpreted the data for the work. GS acquired, analyzed, and interpreted the data for the work. RL-M made substantial contributions to the conception and design of the work, and revised the manuscript. RB made substantial contributions to the analysis and interpretation of data for the work, and revised the manuscript. MB was involved in the study design, helped in writing the manuscript, revised the manuscript, supervised the data collection and interpretation, supervised the postdoc co-authors, approved the content for publication, and is accountable for all aspects of the work.

## Conflict of Interest Statement

The authors declare that the research was conducted in the absence of any commercial or financial relationships that could be construed as a potential conflict of interest.

## Footnotes

1 www.pantherdb.org

2 www.yeastgenome.org

## Supplementary Material

FIGURE S1 WT strain expressing Htt-103Q-GFP alone (YOK 2842) or Htt-103Q-GFP together with *SLX5* (YOK 2843) were grown to mid-logarithmic phase in selective medium. Images of yeast cells with diffuse staining 103Q-GFP, aggregates, and speckles were recorded, counted, and graphed. Additionally, we stained cells with the LIVE/DEAD Yeast Viability Kit (Thermo Fisher) to quantitate dead or dying cells in the culture (dead). Average counts for three independent experiments were graphed +/− standard deviation. Y-axis: percent of cells.

FIGURE S2 The *slx5^SIM^* mutant suppresses lethality of Htt-103Q in *slx5*Δ. *slx5*Δ strain YOK821 expressing 103Q-Htt/URA3 was transformed with *SLX5* plasmid (BOK376), *slx5^SIM^* mutant (BOK463), and an empty vector (pRS425). Resulting transformants were struck to appropriate selective media and incubated for 3 days at 30°C.

FIGURE S3 Steady-state protein levels of AD-25Q and AD-55Q are not grossly affected by expression of BD-Slx5. AD-25Q and AD-55Q were expressed in the presence or absence of BD-Slx5 in the yeast two-hybrid reporter strain. Proteins were extracted, separated by SDS–PAGE and western blotted with an antibody to the Gal4-AD or PGK, a loading control (Szymanski and Kerscher, 2013). EV – empty Gal4-AD vector expressing only Gal4-AD.

TABLE S1 Strains used in this study.

TABLE S2 Expression profiles of 398 genes regulated by an extra copy of *SLX5*. An extra copy of *SLX5* alters the expression profiles of 398 genes in the Htt103Q background (Htt103Q-NLS [Vector] vs. Htt103Q-NLS [*SLX5*]). 363 out of these 398 genes are also found to be differently expressed between Htt25Q-NLS and Htt103Q-NLS (Htt25Q[Vector] vs. Htt103Q[Vector]). Expression of most of the 363 genes (99.4%) is inversely correlation between Htt25Q [Vector] vs. Htt103Q [Vector] and Htt103Q [Vector] vs. Htt103Q [*SLX5*]. Only two genes do not exhibit this inverse relationship (Exceptions: YDL182W and YGR092W). Up- and down-regulated transcripts are shown in red and blue, respectively. Green shows 35 genes that are not detected the comparison between Htt-25Q and Htt-103Q (Htt25Q [Vector] vs. Htt103Q [Vector]).

TABLE S3 List of clustering genes. The 398 genes, differently expressed between Htt103Q [Vector] and Htt103Q [*SLX5*], are analyzed. We used systematic name to search the neighboring genes. Up- and down-regulated profiles are shown in red and blue, respectively.

TABLE S4 Cellular Components Categories of genes modulated by Slx5. The number of individual genes placed into cellular component categories is listed in the table. Individual genes are colored red (increased expression in cells with Htt103Q-NLS and Slx5) or blue (decreased expression in cells with Htt103Q-NLS and Slx5), black (only one data-point – compare **Supplementary Table S2**). Note that some genes analyzed are part of multiple cellular component categories.
