# Supplementary figures and images for: SUMO-Targeted Ubiquitin Ligases (STUbLs) Reduce the Toxicity and Abnormal Transcriptional Activity Associated With a Mutant, Aggregation-Prone Fragment of Huntingtin
Source: Front Genet. 2018 Sep 18;9:379. doi: 10.3389/fgene.2018.00379 (PMC6154015; doi:10.3389/fgene.2018.00379)

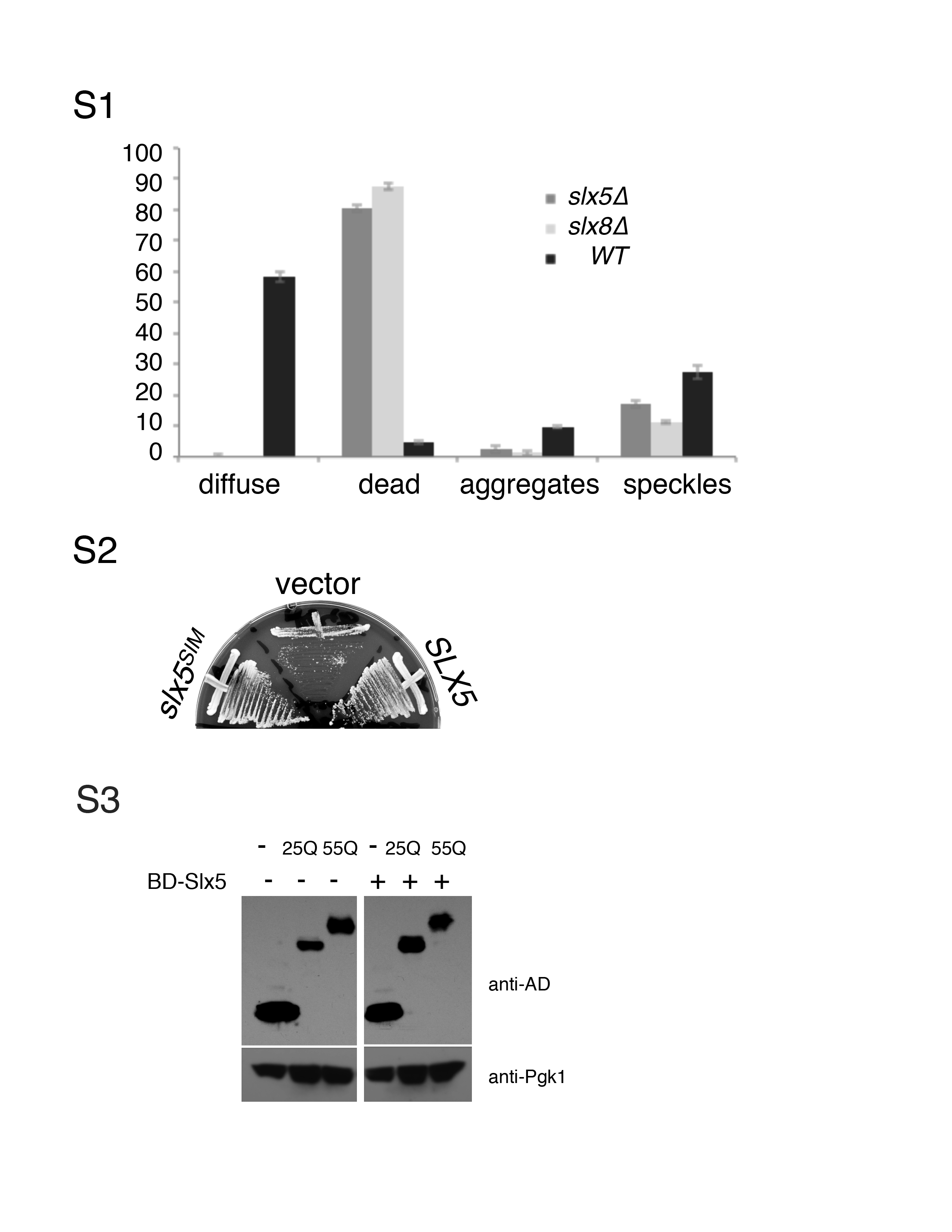

Supplement: FIGURE S1 — WT strain expressing Htt-103Q-GFP alone (YOK 2842) or Htt-103Q-GFP together with SLX5 (YOK 2843) were grown to mid-logarithmic phase in selective medium. Images of yeast cells with diffuse staining 103Q-GFP, aggregates, and speckles were recorded, counted, and graphed. Additionally, we stained cells with the LIVE/DEAD Yeast Viability Kit (Thermo Fisher) to quantitate dead or dying cells in the culture (dead). Average counts for three independent experiments were graphed +/− standard deviation. Y-axis: percent of cells. [file Image_1.TIF]
