# Supplementary material for: SUMO-Targeted Ubiquitin Ligases (STUbLs) Reduce the Toxicity and Abnormal Transcriptional Activity Associated With a Mutant, Aggregation-Prone Fragment of Huntingtin
Source: Front Genet. 2018 Sep 18;9:379. doi: 10.3389/fgene.2018.00379 (PMC6154015; doi:10.3389/fgene.2018.00379)
Supplement: TABLE S1 — Strains used in this study. [file Table_1.DOCX]

| **Name** | **Genotype or strain background** | **Plasmids** | **Reference** |
| --- | --- | --- | --- |
| YOK 820 (MHY501) | *Mat α his3-∆200 leu2-3, 112 ura3-52 lys2-801 trp1-1 gal2* |  | Li and Hochstrasser, 2003 |
| YOK 821  (MHY 3712) | *slx5::KanMX4* in MHY501 |  | Xie et al. 2007 |
| YOK 823  (MHY 3716) | *slx8::KanMX4* in MHY501 |  | Xie et al. 2007 |
| YOK 2206 | YOK 821 | GPD-25QHtt in pRS416 URA3/CEN (BOK 786) | This study |
| YOK 2207 | YOK 821 | GPD-103QHtt in pRS416 URA3/CEN (BOK 785) | This study |
| YOK 2827 | YOK 821 | pRS316 URA3/CEN (BOK 336) | This study |
| YOK 2209 | YOK 820 | GPD-25QHtt in pRS416 URA3/CEN (BOK 786) | This study |
| YOK 2210 | YOK 820 | GPD-103QHtt in pRS416 URA3/CEN (BOK 785) | This study |
| YOK 2828 | YOK 820 | pRS316 URA3/CEN (BOK 336) | This study |
| YOK 2824 | YOK 823 | GPD-103QHtt in pRS416 URA3/CEN (BOK 785) |  |
| YOK 2825 | YOK 823 | GPD-25QHtt in pRS416 URA3/CEN (BOK 786) | This study |
| YOK 2826 | YOK 823 | pRS316 URA3/CEN (BOK 336) | This study |
| YOK 2646 | *slx5::KanMX4* in JD52 | GAL-103Q Htt in pRS416 URA3/CEN (BOK 943) | This study |
| YOK 2647 | *slx5::KanMX4* in JD52 | GAL-25Q Htt in pRS416 URA3/CEN (BOK 942) | This study |
| YOK 2648 | JD52 WT | GAL-103Q Htt in pRS416 URA3/CEN (BOK 943) | This study |
| YOK 2649 | JD52 WT | GAL-25Q Htt in pRS416 URA3/CEN (BOK 942) | This study |
| YOK 2650 | JD52 WT | GAL-103Q Htt in pRS416 URA3/CEN (BOK 943) and SLX5 in pRS313 HIS3/CEN (BOK 372) | This study |
| YOK 3112 | JD52 WT | GAL-97Q Htt-DsRED TRP1/CEN (BOK 1213) | This study |
| YOK 3023 | pJ694 alpha | Empty vector-BD/TRP (BOK 313) and 25Q Htt-AD/LEU2 (BOK 1207) | This study |
| YOK 3025 | pJ694 alpha | Empty vector-BD (BOK 313) and 55Q Htt-AD/LEU2 (BOK 1209) | This study |
| YOK 3020 | pJ694 alpha | SLX5-BD/TRP1 (BOK 293) and Empty vector-AD/LEU2 (BOK 1137) | This study |
| YOK 3017 | pJ694 alpha | SLX5-BD/TRP1 (BOK 293) and 25Q Htt-AD/LEU2 (BOK 1207) | This study |
| YOK 3014 | pJ694 alpha | SLX5-BD/TRP1 (BOK 293) and 55Q Htt-AD/LEU2 (BOK 1209) | This study |
| YOK 736 | pJ694 alpha | SMT3-BD/TRP (BOK 295) and Slx5-AD/LEU2 (BOK 290) | This study |
| YOK 3064 | pJ694 alpha | SLX5-sim**-BD/TRP1 (BOK 627) and 55Q Htt-AD/LEU2 (BOK 1209) | This study |
| YOK 3072 | pJ694 alpha | RNF4-BD/TRP1 (BOK 556) and 25Q Htt-AD/LEU2 (BOK 1207) | This study |
| YOK 3073 | pJ694 alpha | RNF4-BD/TRP1 (BOK 556) + 55Q-AD/LEU2 (BOK 1209) | This study |
| YOK 3080 | pJ694 alpha | Empty vector-BD/TRP1 (BOK 313) and 55Q Htt-snm*-AD/LEU2 (BOK 1209) | This study |
| YOK 3082 | pJ694 alpha | SLX5-BD/TRP1 (BOK 293) and 55Q Htt-snm*-AD/LEU2 (BOK 1209) | This study |
| YOK 3084 | pJ694 alpha | SLX5-sim**-BD/TRP1 (BOK 627) and 55Q Htt-snm*-AD/LEU2 (BOK 1209) | This study |
| YOK3455 | W303 integrant | *GAL-NLS-Htt25Q-GFP::LEU2* | This study |
| YOK3457 | W303 integrant | *GAL-NLS-Htt103Q-GFP::LEU2* | This study |
| YMB10289 | YOK3457 with BOK333 | *GAL-NLS-Htt103Q-GFP::LEU2 with empty vector* | This study |
| YMB10290 | YOK3457 with BOK372 | *GAL-NLS-Htt103Q-GFP::LEU2 with SLX5/CEN/HIS* | This study |
| YMB10544 | YOK3455 with BOK333 | *GAL-NLS-Htt25Q-GFP::LEU2 with empty vector* | This study |
| YMB10545 | YOK3455 with BOK372 | *GAL-NLS-Htt25Q-GFP::LEU2 with SLX5/CEN/HIS* | This study |
