# Supplementary material for: SUMO-Targeted Ubiquitin Ligases (STUbLs) Reduce the Toxicity and Abnormal Transcriptional Activity Associated With a Mutant, Aggregation-Prone Fragment of Huntingtin
Source: Front Genet. 2018 Sep 18;9:379. doi: 10.3389/fgene.2018.00379 (PMC6154015; doi:10.3389/fgene.2018.00379)
Supplement: TABLE S4 — Cellular Components Categories of genes modulated by Slx5. The number of individual genes placed into cellular component categories is listed in the table. Individual genes are colored red (increased expression in cells with Htt103Q-NLS and Slx5) or blue (decreased expression in cells with Htt103Q-NLS and Slx5), black (only one data-point – compare Supplementary Table S2). Note that some genes analyzed are part of multiple cellular component categories. [file Table_4.pdf]

Selected Components

Cellular Component	Genes	Counts	Count of Overexpressed	Count of Underexpressed	No Data on expression
Cytoplasm or Cytosol	GTR2/YGR163W, GSP2/YOR185C, YNL194C,RSM27/YGR215W, ICT1/YLR099C, ZDS2/YML109W,TSL1/YML100W, LYS20/YDL182W, FMP45/YDL222C, ALG6/YOR002W, NHP2/YDL208W, YDL124W, SHH4/YLR164W, TPS2/YDR074W, COA2/YPL189C-A[3], GAL1/YBR020W, CAT5/YOR125C, FIS1/YIL065C, PRR2/YDL214C, COS2/YML132W, BEM2/YER155C, VMR1/YHL035C, ARG1/YOL058W, GRH1/YDR517W, SSA2/YLL024C, NCA2/YPR155C, YBR139W, RPS27A/YKL156W, XKS1/YGR194C, MRPL49/YJL096W, GYS1/YFR015C, MCH4/YOL119C, MET16/YPR167C, PDH1/YPR002W, ISA2/YPR067W, TAH18/YPR048W, OPI1/YHL020C, GAD1/YMR250W, YGL185C, PET191/YJR034W, TMA22/YJR014W, YOS1/YER074W-A, MIX17/YMR002W, PGM2/YMR105C, MMM1/YLL006W, GPH1/YPR160W, LEU9/YOR108W, ATP17/YDR377W, ATG7/YHR171W, MDE1/YJR024C, EMC6/YLL014W, ERG2/YMR202W, ERP5/YHR110W, RPL7A/YGL076C, EOS1/YNL080C, YPR1/YDR368W, YJR096W, DCS2/YOR173W, BCH1/YMR237W, CTT1/YGR088W, YDJ1/YNL064C, FMP46/YKR049C, HEM13/YDR044W, CNL1/YDR357C, SYN8/YAL014C, PRM8/YGL053W, COS5/YJR161C, GTO3/YMR251W, ECM4/YKR076W, CDC10/YCR002C, GSY2/YLR258W, COS3/YML132W, SYM1/YLR251W, UBP15/YMR304W, RPB7/YDR404C, SKN1/YGR143W, SAH1/YER043C, JAC1/YGL018C, GLC3/YEL011W, AGX1/YFL030W, CLB1/YGR108W, ARL3/YPL051W, RPA43/YOR340C, GOR1/YNL274C, LSP1/YPL004C, SOL4/YGR248W, GRE3/YHR104W, PET100/YDR079W, SEC11/YIR022W, ADE3/YGR204W, ZTA1/YBR046C[2], ERP3/YDL018C, TLG1/YDR468C, ADO1/YJR105W, SNO2/YNL334C, YPT53/YNL093W, RPL7B/YPL198W, GLK1/YCL040W, GCY1/YOR120W, RTC3/YHR087W, FUN14/YAL008W, TKL2/YBR117C, MSW1/YDR268W, UPS2/YLR168C, COS8/YHL048W, STR3/YGL184C, YNL200C, YMR196W, YPR172W, YKL023W, YOR289W, YER134C, YML131W, YNR034W-A, YKR045C, YDR391C, YOR111W, YKR041W, RAP1/YNL216W, PPA2/YMR267W, VID30/YGL227W, SEN1/YLR430W, GDB1/YPR184W, TFS1/YLR178C, MET30/YIL046W, SAN1/YDR143C, PCL8/YPL219W, YNK1/YKL067W, MSD1/YPL104W, RNY1/YPL123C, GRX7/YBR014C, ALG13/YGL047W, RFS1/YBR052C, ARO9/YHR137W, NTO1/YPR031W, RBG2/YGR173W, MOB2/YFL034C-B, NQM1/YGR043C, MET3/YJR010W, NTH1/YDR001C, ETP1/YHL010C, CRG1/YHR209W, ALD3/YMR169C, MSB1/YOR188W[1,3], MMS2/YGL087C, TPK1/YJL164C, PHR1/YOR386W, IQG1/YPL242C, FKH1/YIL131C, HST2/YPL015C, MAP1/YLR244C, YCP4/YCR004C, HSP31/YDR533C, NCE102/YPR149W, SCS22/YBL091C-A, GND2/YGR256W, GRX1/YCL035C, NTH2/YBR001C, TAD2/YJL035C, AMD1/YML035C, SDS23/YGL056C, MET6/YER091C, RCK2/YLR248W, GLO2/YDR272W, RKM3/YBR030W, BAT2/YJR148W, ATF2/YGR177C, UGP1/YKL035W, SDP1/YIL113W, GPP2/YER062C, DOG2/YHR043C, PIG2/YIL045W, PHO4/YFR034C, GPX1/YKL026C, LYS1/YIR034C, UBC11/YOR339C, SLM5/YCR024C, SSP120/YLR250W, ALD6/YPL061W, YAK1/YJL141C, TSR4/YOL022C, GRX2/YDR513W, YCH1/YGR203W, TSA2/YDR453C, AIM10/YER087W, DBF2/YGR092W, REF2/YDR195W, RTS3/YGR161C, ECM25/YJL201W, SFG1/YOR315W, ZRG8/YER033C, ATG34/YOL083W, SLM4/YBR077C, MNE1/YOR350C, RTN2/YDL204W, PAI1/YMR174C, STF2/YGR008C, DDR48/YMR173W, EAF5/YEL018W, MEH1/YKR007W, PEX21/YGR239C, MDR1/YGR100W, GYP5/YPL249C, YMR090W, CAP2/YIL034C, VID24/YBR105C, UFO1/YML088W, GIP2/YER054C, ADP1/YCR011C, TGL3/YMR313C, AIM20/YIL158W, THI4/YGR144W	208	142	52	14
Nucleus (inlcuding all nuclear parts i.e. nucleolous)	GSP2/YOR185C, MED4/YOR174W, TFA2/YKR062W,NHP2/YDL208W, KDX1/YKL161C, PRR2/YDL214C, COS2/YML132W, SSA2/YLL024C, SRB7/YDR308C, OPI1/YHL020C, YGL185C, MCM1/YMR043W, MIX17/YMR002W, RPP1/YHR062C, DBF4/YDR052C, DCS2/YOR173W, SNF5/YBR289W, RAD2/YGR258C, ABD1/YBR236C, BUD27/YFL023W, RPB7/YDR404C, CLB1/YGR108W, RPA43/YOR340C, GOR1/YNL274C, YAP7/YOL028C, ADO1/YJR105W, RTC3/YHR087W, TKL2/YBR117C, BUD22/YMR014W[3], YMR310C, YCR087C-A, YPR172W, YDR018C, YOR289W, YER134C, YKR075C, YDR391C, YKL091C, RAP1/YNL216W, SRS2/YJL092W, HMX1/YLR205C, VID30/YGL227W, SEN1/YLR430W, MET30/YIL046W, SAN1/YDR143C, PCL8/YPL219W, NMA2/YGR010W, ALG13/YGL047W, ARO9/YHR137W, NTO1/YPR031W, MOB2/YFL034C-B, NQM1/YGR043C, NAF1/YNL124W, MMS2/YGL087C, TPK1/YJL164C, PHR1/YOR386W, FKH1/YIL131C, HST2/YPL015C, GRX1/YCL035C, TAD2/YJL035C, SDS23/YGL056C, RKM3/YBR030W, BAT2/YJR148W, HAP3/YBL021C, PSY2/YNL201C, SDP1/YIL113W, GPP2/YER062C, DOG2/YHR043C, PHO4/YFR034C, CFT2/YLR115W, UBC11/YOR339C, YAK1/YJL141C, HMI1/YOL095C, GRX2/YDR513W, YCH1/YGR203W, DBF2/YGR092W, SLX5/YDL013W, KRE33/YNL132W, REF2/YDR195W, PSH1/YOL054W, RTS3/YGR161C, ESC8/YOL017W, ABF1/YKL112W, IML3/YBR107C, SFG1/YOR315W, snR86, ASI1/YMR119W, LIP1/YMR298W, YPR158C-D , SPO12/YHR152W, YOR192C-B, UIP4/YPL186C, YER138C, ESC2/YDR363W, YAR010C, YPR158W-B, SYC1/YOR179C, YPR158C-C, RTN2/YDL204W, PAI1/YMR174C, EAF5/YEL018W, CBC2/YPL178W, UFO1/YML088W, SPT20/YOL148C, GAL11/YOL051W, RRP9/YPR137W, AIM20/YIL158W	108	58	41	9
Mitochondria	RSM27/YGR215W, ICT1/YLR099C, LYS20/YDL182W,SHH4/YLR164W, COA2/YPL189C-A, CAT5/YOR125C, FIS1/YIL065C, NCA2/YPR155C, MRPL49/YJL096W, PDH1/YPR002W, ISA2/YPR067W, PET191/YJR034W, MIX17/YMR002W, MMM1/YLL006W, LEU9/YOR108W, ATP17/YDR377W, FMP46/YKR049C, JAC1/YGL018C, PET100/YDR079W, YPT53/YNL093W, FUN14/YAL008W, MSW1/YDR268W, UPS2/YLR168C, YNL200C, YPR098C, SCO2/YBR024W, AIM41/YOR215C, MRF1/YGL143C, YPL109C, LYS4/YDR234W, YNL122C, YKL133C, YNL195C, YKR041W, CHA1/YCL064C, MRX10/YDR282C, PPA2/YMR267W, GDB1/YPR184W, NDE2/YDL085W, MNR2/YKL064W, YNK1/YKL067W, BIO2/YGR286C, MSD1/YPL104W, FMP25/YLR077W, MRH1/YDR033W, FMP45/YNL168C, MET3/YJR010W, RCF1/YML030W, ADL4/YOR374W, AIM17/YHL021C, FSF1/YOR271C, MSB1/YOR188W[1,3], PHR1/YOR386W, YCP4/YCR004C, YRO2/YBR054W, NCE102/YPR149W, NTH2/YBR001C, CEM1/YER061C, BAT2/YJR148W, FMP27/YLR454W, GPX1/YKL026C, SLM5/YCR024C, OSM1/YJR051W, ALD6/YPL061W, HMI1/YOL095C, GRX2/YDR513W, AIM10/YER087W, MGR1/YCL044C, MRPL50/YNR022C, ZRG8/YER033C, UIP4/YPL186C, INA17/YPL099C, CBT1/YKL208W, MSC1/YML128C, OM45/YIL136W, SPG1/YGR236C, MNE1/YOR350C, STF1/YDL130W-A, FMP33/YJL161W, FMP16/YDR070C, TAZ1/YPR140W	81	67	10	4
Endoplasmic Reticulum	ALG6/YOR002W, OPI1/YHL020C, YOS1/YER074W-A, MMM1/YLL006W, EMC6/YLL014W, ERG2/YMR202W, ERP5/YHR110W, EOS1/YNL080C, PRM8/YGL053W, SKN1/YGR143W, SEC11/YIR022W, ERP3/YDL018C, YPT53/YNL093W, YDR018C, YNL058C, HMX1/YLR205C, ALG13/YGL047W, NCR1/YPL006W, YRO2/YBR054W, NCE102/YPR149W, SCS22/YBL091C-A, ERG5/YMR015C[3], HNM1/YGL077C, TDA4/YJR116W, ATF2/YGR177C, ERG11/YHR007C, OSM1/YJR051W, ALG3/YBL082C, SMF3/YLR034C, LIP1/YMR298W, UIP4/YPL186C, MSC1/YML128C, SPG1/YGR236C, RTN2/YDL204W, YDR098C-A, ADP1/YCR011C, PHM7/YOL084W	37	27	10	0
Peroxisome	FIS1/YIL065C, AGX1/YFL030W, GPX1/YKL026C, PEX21/YGR239C	4	3	0	1
Golgi Apparatus	GRH1/YDR517W, YOS1/YER074W-A, ERP5/YHR110W, BCH1/YMR237W, PRM8/YGL053W, ARL3/YPL051W, ERP3/YDL018C, YPT53/YNL093W, GRX7/YBR014C, VTH2/YJL222W, AKR1/YDR264C, GNT1/YOR320C	12	11	1	0
Ribosome	ALG6/YOR002W, RPS27A/YKL156W, MRPL49/YJL096W, TMA22/YJR014W, RPL7A/YGL076C, RPL7B/YPL198W, YNL122C, RPL43A/YPR043W	8	4	3	1
Cell Wall	MKC7/YDR144C, SPI1/YER150W, YPS6/YIR039C, CWP1/YKL096W, PIR3/YKL163W, TIR4/YOR009W, PAU24/YBR301W, UTR2/YEL040W, TIR1/YER011W[3], DIA3/YDL024C	10	5	4	1
Vacuole	VMR1/YHL035C, YBR139W, MCH4/YOL119C, YPT53/YNL093W, YKR075C, YNL115C, YNL058C, YBR241C, RNY1/YPL123C, APE3/YBR286W, NCR1/YPL006W, GNT1/YOR320C, SMF3/YLR034C, PHM6/YDR281C, PAU10/YDR542W, YMR244W	16	11	4	1
Plasma Membrane	FLO11/YIR019C, SLM2/YNL047C, PNS1/YOR161C, PMC1/YGL006W, YNL194C, HXT10/YFL011W, HOL1/YNR055C, FMP45/YDL222C, MKC7/YDR144C, VMR1/YHL035C, SSA2/YLL024C, YPS6/YIR039C, QDR3/YBR043C, STE3/YKL178C, PRM8/YGL053W, HXT5/YHR096C, STL1/YDR536W, LSP1/YPL004C, ADP1/YCR011C, KIN82/YCR091W, YPT53/YNL093W, YBL029C-A, HBT1/YDL223C, CCH1/YGR217W	24	15	7	2
Extracellular Region	YJL132W, YBR053C, PPA2/YMR267W, RNY1/YPL123C, MRH1/YDR033W, TIR4/YOR009W, PAU24/YBR301W, UTR2/YEL040W, MET6/YER091C, TIR1/YER011W[3], FLO11/YIR019C, YGP1/YNL160W,	12	5	5	2
Unlikely to Encode Functional Protein (Dubious ORF)	YER066C-A, YJL009W, YKL053W, YPL185W, YPL114W, YNL114C, YNL170W, YGR293C, YPR126C, YDL011C, YGR190C, YOR121C, YGL199C, YKL177W, YNL057W, YLR349W, YFL013W-A, YER088C-A, YLR101C, YNR025C, YOL046C, YDR426C, YDL050C, YDL023C, YKL030W, YOR248W, YLR252W, YPR142C, YER133W-A, YPL182C, YCL007C, YMR173W-A	32	10	13	9
Unknown	YKL068W-A, YIR042C, YMR181C, YOL029C, YDR461C-A, YNL193W, YDL177C, YCR022C, EMI1/YDR512C, AYT1/YLL063C, GLG1/YKR058W, MTC7/YEL033W, UGX2/YDL169C, YPS63/YLR261C, RIM13/YMR154C, GPG1/YGL121C, YGR066C, ERR1/YPL281C, KIN82/YCR091W, YDL057W, YBL081W, YLR279W, YNL033W, YOR097C	24	15	6	3
**Totals**	**Note: totals indicate unique genes listed in the table above**	398	248	115	35

KEY:
Underexpressed
Overexpressed
No data on expression

Previously associated with Huntington's
[1] Muchowski science 2003
[2] Panther
[3] Wolfe et. al 2014

Counts

300
